# Seroprevalence of SARS-CoV-2 Antibodies Among Children in School and Day Care in Montreal, Canada

**DOI:** 10.1001/jamanetworkopen.2021.35975

**Published:** 2021-11-23

**Authors:** Kate Zinszer, Britt McKinnon, Noémie Bourque, Laura Pierce, Adrien Saucier, Alexandra Otis, Islem Cheriet, Jesse Papenburg, Marie-Ève Hamelin, Katia Charland, Julie Carbonneau, Monica Zahreddine, Ashley Savard, Geneviève Fortin, Alexander Apostolatos, Nancy Haley, Nathalie Ratté, Isabel Laurin, Cat Tuong Nguyen, Patrica Conrod, Guy Boivin, Gaston De Serres, Caroline Quach

**Affiliations:** 1 University of Montreal, Montreal, Quebec, Canada; 2 Centre for Public Health Research, Montreal, Quebec, Canada; 3 University of Toronto, Toronto, Ontario, Canada; 4 Montreal Children’s Hospital, Montreal, Quebec, Canada; 5 McGill University Health Centre, Montreal, Quebec, Canada; 6 Research Centre Laval University, Quebec City, Quebec, Canada; 7 Direction régionale de la santé publique du Centre intégré universitaire de santé et de services sociaux du Centre-Sud-de-l’Île-de-Montréal, Montreal, Quebec, Canada; 8 Research Centre of the Sainte-Justine University Hospital, Montreal, Quebec, Canada; 9 National Institute of Public Health of Quebec, Quebec City, Quebec, Canada

## Abstract

**Importance:**

Quebec prioritized in-person learning after the first wave of the COVID-19 pandemic, with school closures being implemented temporarily in selected schools or in hot-spot areas. Quebec’s decision to keep most schools open was controversial, especially in Montreal, which was the epicenter of Canada’s first and second waves; therefore, understanding the extent to which children were infected with SARS-CoV-2 provides important information for decisions about school closures.

**Objective:**

To estimate the seroprevalence of SARS-CoV-2 antibodies in children and teenagers in 4 neighborhoods of Montreal, Canada.

**Design, Setting, and Participants:**

This cohort study (the Enfants et COVID-19: Étude de séroprévalence [EnCORE] study) enrolled a convenience sample of children aged 2 to 17 years between October 22, 2020, and March 22, 2021, in Montreal, Canada.

**Exposures:**

Potential exposure to SARS-CoV-2.

**Main Outcomes and Measures:**

The main outcome was seroprevalence of SARS-CoV-2 antibodies, collected using dried blood spots (DBSs) and analyzed with a research-based enzyme-linked immunosorbent assay (ELISA). Parents also completed an online questionnaire that included questions on self-reported COVID-19 symptoms and tests, along with sociodemographic questions.

**Results:**

This study included 1632 participants who provided a DBS sample from 30 day cares, 22 primary schools, and 11 secondary schools. The mean (SD) age of the children who provided a DBS sample was 9.0 (4.4) years; 801 (49%) were female individuals, with 354 participants (22%) from day cares, 725 (44%) from primary schools, and 553 (34%) from secondary schools. Most parents had at least a bachelor’s degree (1228 [75%]), and 210 (13%) self-identified as being a racial or ethnic minority. The mean seroprevalence was 5.8% (95% CI, 4.6%-7.0%) but increased over time from 3.2% (95% CI, 0.7%-5.8%) in October to November 2020 to 8.4% (95% CI, 4.4%-12.4%) in March to April 2021. Of the 95 children with positive SARS-CoV-2 antibody results, 78 (82%) were not tested or tested negative with reverse transcription–polymerase chain reaction (RT-PCR) testing, and all experienced mild (49 [52%]) or no clinical symptoms (46 [48%]). The children of parents who self-identified as belonging to a racial and ethnic minority group were more likely to be seropositive compared with children of White parents (adjusted seroprevalence ratio, 1.9; 95% CI, 1.1-2.6).

**Conclusions and Relevance:**

These results provide a benchmark of the seroprevalence status in Canadian children. The findings suggest that there was more transmission occurring in children compared with what was being detected by RT-PCR, although children experienced few or mild symptoms. It will be important to continue monitoring the serological status of children, particularly in the context of new COVID-19 variants of concern and in the absence of mass vaccination campaigns targeting young children.

## Key Points

**Question:**

What is the seroprevalence of SARS-CoV-2 antibodies in a convenience sample of children aged 2 to 17 years in Montreal, Canada, enrolled between October 2020 and March 2021?

**Findings:**

In this cohort study of 1632 participants, the mean baseline seroprevalence of SARS-CoV-2 was 5.8%. Of the 95 participants who were seropositive for SARS-CoV-2 antibodies, 82% were not tested or tested negative, and all experienced either mild or no clinical symptoms.

**Meaning:**

The findings suggest that there was more transmission occurring in children compared with what was being detected, although children experienced few or mild symptoms.

## Teaser

This cohort study estimates the seroprevalence of SARS-CoV-2 antibodies in children and teenagers in 4 neighborhoods of Montreal, Canada.

## Introduction

Children and adolescents are susceptible to infection with SARS-CoV-2 but experience much lower rates of severe COVID-19 disease than adults.^1^ Many children experience no or mild symptoms of infection, and evidence prior to the emergence of variants of concern (VOC) suggests young children probably played a smaller role in transmission than adults,^2,3^ while older children and adolescents may have transmission rates more similar to adults.^3^ The role children play in transmission of the virus has been the topic of much debate, especially with respect to the safety of in-person schooling.^4^

Through the first year of the SARS-CoV-2 pandemic, hundreds of millions of students around the world were affected by the closures of schools and day cares.^5^ Some countries have kept schools mostly open (eg, Sweden, Taiwan), while others have opted for extended closures (eg, US, India). In Canada, schools were mostly closed during the first few months of the pandemic (March to June 2020), but have opened for in-person learning on a province-by-province basis during the September 2020 to June 2021 school year. Despite having one of the highest provincial incidence and mortality rates, Quebec has prioritized in-person learning, with school closures being implemented temporarily in selected schools or in hot-spot areas such as Quebec City.^6^ Quebec’s decision to keep most schools open has remained controversial, especially in Montreal, which was the epicenter of Canada’s first and second waves.^7,8^

Longitudinal seroprevalence studies conducted in schools and day cares are beginning to advance our knowledge of SARS-CoV-2 transmission among children, staff, and families in educational settings. For example, studies conducted in French^9^ and British primary schools and French day cares^10^ suggested little evidence of transmission from children to their peers or teachers, and a large Swiss cohort^11^ found minimal clustering of seropositive cases within classes and schools, despite a clear increase in seroprevalence in children over a 5-month period of very high transmission. Determinants of SARS-CoV-2 seropositivity among children have included contact with a confirmed COVID-19 case,^10,12^ health care workers in the household,^12^ belonging to a minority racial or ethnic group, and geographical area.^11^ One year into the pandemic, little is known regarding the seroprevalence of SARS-CoV-2 infection and its determinants among children and adolescents in Canada.

This article reports baseline seroprevalence results from wave 1 (October 2020 to April 2021) of the Enfants et COVID-19: Étude de séroprévalence (EnCORE^13^) study, a longitudinal cohort study in primary and secondary schools and day cares in Montreal. In addition, we examined associations between seroprevalence and sociodemographic and household characteristics, reported COVID-19 symptoms and tests, and potential COVID-19 risk factors and prevention efforts.

## Methods

### Study Design and Conduct

This study is a cohort study based on 4 sentinel neighborhoods from the 34 boroughs and independent municipalities that make up the island of Montreal (population, 1.9 million people^14^). These neighborhoods were selected to reflect diversity in terms of geography, cumulative COVID-19 cases, and neighborhood socioeconomic status.^15^ The populations living in the West Island and Plateau-Mont-Royal are more affluent and highly educated, while Montreal North is one of the city’s poorest and most racially diverse neighborhoods, and Mercier-Hochelaga-Maisonneuve is a working-class neighborhood with nearly one-third of the population living below the poverty line.^16^ Schools and day cares within each neighborhood were selected by the research team and school boards to participate in this study. The study team provided a letter of invitation for parents that was sent either by the school board or by the participating school or day care. The letter of invitation was emailed a maximum of 3 times to parents.

Ethics approval was received from the research ethics boards of the Université de Montréal and the Centre Hospitalier Universitaire Sainte-Justine. Details of the full cohort study procedures are available in the study protocol.^15^ This study followed the reporting requirements of the Strengthening the Reporting of Observational Studies in Epidemiology (STROBE) statement.^17^

### Eligibility Criteria and Study Procedure

Participants were eligible for enrollment if they attended 1 of the participating schools or day cares, if they were between the ages of 2 and 17 years, and if their parent or legal guardian gave informed consent to participate and the child gave their assent. Multiple children per household were eligible, as long as they all attended a participating school or day care. Parental consent and their child’s assent were provided online, which was followed by an online questionnaire. Once the baseline questionnaire was complete, an at-home dried blood spot (DBS) collection kit was mailed to their residence.

The questionnaire collected the following information from parents: dates and results of prior reverse transcriptase–polymerase chain reaction (RT-PCR) tests and the presence of SARS-CoV-2 symptoms and the time of RT-PCR tests; dates of hospitalizations and COVID-19 vaccinations for the participant and household members; basic sociodemographic and household characteristics; preventive behaviors in the household and at school and/or day care; and health and lifestyle information for the child. Presently, there are 2 waves of data collection planned for spring and fall 2021.

### Serological Samples and Laboratory Analysis

Specimen collection kits for finger prick whole blood samples were sent to the participant’s household 1 day after completion of the online questionnaire. Kits contained printed directions for collection, storage, and mailing for each specimen. Participants also had access to tutorial videos explaining each procedure step-by-step, and video conference support from the research team was available on request. Parents used contact-activated retractable lancets (BD Microtainer) and a Whatman 903 protein saver card for their child’s DBS collection. They were asked to dry the samples for at least 2 hours and mail them in a protective envelope with desiccant within 24 hours of collection. On reception in the laboratory, the samples were analyzed for quality control purposes, such as sufficient quantity of blood or layering of blood drops. For the samples that did not pass the quality control, participants and their parents were asked to redo the sample collection. The filter papers with DBS were conserved at −20 °C, and blood was eluted overnight the day before the serological assay. Samples were processed in 96-well plates with 40 samples from participants and 7 control samples (positive and negative) per plate.

The serostatus of participants was determined by an enzyme-linked immunosorbent assay (ELISA) that we developed and validated using the receptor-binding domain (RBD) from the spike protein as an antigen. Prior to beginning the study, we successfully validated the ELISA assay with positive control samples (participants with RT-PCR–confirmed SARS-CoV-2 infection and known to be seropositive for anti–SARS-CoV-2 antibodies) and negative control samples (SARS-CoV-2 seronegative). Based on the results, the assay had a sensitivity of 95% and specificity of 100%. A colorimetric reaction determined by optic density (OD) allowed the detection of IgG in the samples and the evaluation of the signal generated by SARS-CoV-2–specific antibodies against the RBD antigen confirmed whether subjects were seropositive. The OD cutoff for positivity was determined based on the average of OD from negative sera plus 3 SDs. Three samples with an indeterminate result (defined as a good quality sample near the threshold for positive/negative) and 33 with a borderline negative result (defined as a poor quality sample analyzed and found to be negative) were classified as seronegative for the primary analysis. Parents were sent an email that provided their child’s serology result and information about what the result means.

### Statistical Analysis

We calculated that a sample size of 457 children per neighborhood (total of 1828 children) would enable estimation of neighborhood-specific seroprevalence with 2% precision.^18^ This was based on a projected seroprevalence of 5%.

The main outcome was seroprevalence, and we calculated descriptive statistics and univariate associations of seroprevalence with sociodemographic and household characteristics, self-reported COVID-19 symptoms and potential COVID-19 risk factors, and prevention efforts. Previous COVID-19 diagnoses via RT-PCR were self-reported by parents. Higher risk conditions were self-reported and included, eg, diabetes, immunocompromised, serious heart conditions, chronic obstructive pulmonary disease, kidney disease, and cancer. Based on Canadian Census categorization, parents were asked whether they self-identify as Arab, Black, Chinese, Filipino, Japanese, Korean, Latin American, South Asian, Southeast Asian, West Asian, and/or White. Due to small numbers within specific racial and ethnic groups, we generated a binary variable (racial or ethnic minority group vs White). Body mass index (BMI) categories were calculated using Stata’s zbmicat function,^19^ which uses BMI cutoffs recommended by the Childhood Obesity Working Group of the International Obesity Taskforce. Other essential occupations included day care educator or worker, corrections or prison officer, teacher or other school staff, first responder, public transportation driver, food service industry, grocery store staff, pharmacy staff, hairdresser or barber, aesthetician, flight attendant, and factory worker. Adjusted seroprevalence differences and ratios by setting of attendance (ie, day care, primary school, secondary school), parental education level, and parental race and ethnicity were estimated by calculating average marginal effects from multivariable logistic regression models, which adjusted for age, sex, neighborhood, and timing of the DBS.

Sensitivity analyses were performed to generate seroprevalence estimates under scenarios in which (1) indeterminate samples were categorized as positive and (2) all indeterminate and borderline negative results were excluded. All analyses used robust standard errors to account for clustering by school and were conducted using Stata version 14 (StataCorp). Statistical significance was set at *P* < .05, and all tests were 2-tailed.

## Results

### Descriptive Characteristics

**Figure 1.**
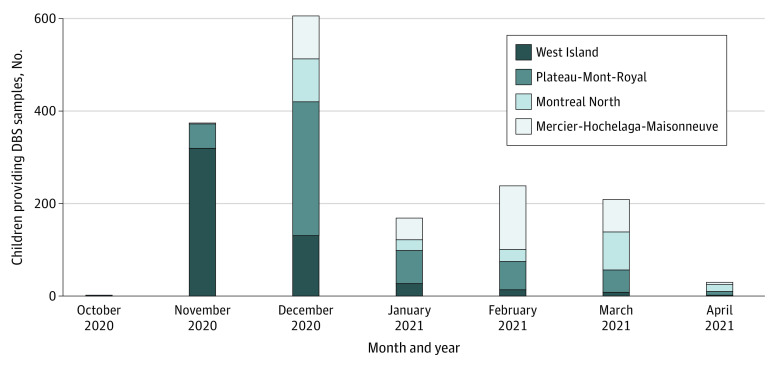
Monthly Participant Recruitment by Neighborhood DBS indicates dried blood spot.

Recruitment followed the approvals of the ethics committees and the school boards; it began in West Island (and surrounding area) on October 16, 2020, followed by Plateau, Mercier-Hochelaga-Maisonneuve, and Montreal North (Figure 1). By the end of the recruitment (March 14, 2021), 30 day cares, 22 primary schools, and 11 secondary schools were participating in the study. The median sizes of the daycares, primary schools, and secondary schools were 90 (range, 46-219), 341 (range, 101-644), and 795 (range, 319-2193) students, respectively. There was a total of 21 564 children who attended the participating day cares or schools, with 20 928 aged between 2 and 17 years who were eligible for inclusion. 1901 children (9%) from 1444 households enrolled in the study and completed the questionnaire. Of these participants, 1632 (86%) provided a DBS sample that was of sufficient quality for the serological analysis. Participation rates varied substantially among the different day care centers and schools (median [IQR], 10% [5%-20%]). Participation rates were lower among parents of secondary school children (7%) compared with day care (14%) and primary school (14%) children. Primary and secondary schools that ranked in the upper half of the school-based socioeconomic distribution also had higher participation rates (24%) vs those in the lower half (5%). 

The mean (SD) age of the children who provided a DBS sample was 9.0 (4.4) years, and 801 (49%) were female individuals, with 354 participants (22%) from day cares, 724 (44%) from primary schools, and 553 (34%) from secondary schools. The planned sample size of 457 was attained for the 2 more affluent neighborhoods (West Island, 504 participants; Plateau-Mont-Royal, 533 participants), but not for Montreal North (237 participants) or Mercier-Hochelaga-Maisonneuve (357 participants). Most parents had at least a bachelor’s degree (1228 [75%]), and 210 (13%) self-identified as belonging to a racial or ethnic minority groups. On average, there were 2 adults and 2 children in 3 to 4–bedroom households. Among the RT-PCR test results reported by participants, 26 were positive prior to DBS collection, and the only significantly different symptom, compared with RT-PCR negative tests (881 participants), was loss of smell (eFigure 1 in the Supplement). All 95 participants with seropositive results either experienced no symptoms (46 [48%]) or mild symptoms (49 [52%]). The median (IQR) time between an RT-PCR positive test result and the DBS collection date was 109 (66-146) days. Two children were hospitalized with or due to COVID-19.

### Seropositivity of SARS-CoV-2

We identified 95 seropositive children out of a total of 1632 children tested, resulting in estimated crude seroprevalence of 5.8% (95% CI, 4.6%-7.0%). This estimate included 36 borderline negative or indeterminate samples that were classified as seronegative. If we excluded these 36 results, the estimated seroprevalence was 6.0% (95% CI, 4.7%-7.2%), and if we assumed the 3 indeterminate samples were instead seropositive, the estimate was 6.1% (95% CI, 4.8%-7.4%).

The seropositive children were from 12 day cares, 15 elementary schools, and 10 secondary schools. Individual and household characteristics associated with seroprevalence are summarized in Table 1. The largest difference in seroprevalence between participants was having had a positive RT-PCR test (prevalence difference, 59.9 percentage points; 95% CI, 39.6-80.2 percentage points) and someone in the household who had a positive RT-PCR test (prevalence difference, 27.4 percentage points; 95% CI, 15.2-39.6 percentage points). Other factors significantly associated with seroprevalence included parental race and ethnicity and neighborhood. The seroprevalence difference for children was 5.8 percentage points (95% CI, 1.3-10.2 percentage points) for children whose parents belonged to racial and ethnic minority groups compared with White parents.

**Table 1.** Crude Seroprevalence and Seroprevalence Differences and Ratios for Children Aged 2 to 17 Years by Individual and Household Characteristics, October 2020 to March 2021 in Montreal, Canada

Demographic characteristics	Children, No./total No. (%)		Seroprevalence, % (95% CI)	Crude prevalence ratio (95% CI)
	Seronegative	Seropositive		
Total	1537	95	5.8 (4.6 to 7.0)	NA
Sex				
Female	746/1537 (49)	55/95 (58)	6.9 (4.9 to 8.8)	1 [Reference]
Male	797/1537 (51)	40/95 (42)	4.8 (3.2 to 6.4)	0.7 (0.4 to 1.0)
Age, y				
2-4	316/1529 (21)	16/95 (17)	4.8 (2.9 to 6.7)	1 [Reference]
5-9	515/1529 (34)	27/95 (28)	5.0 (3.4 to 6.6)	1.0 (0.5 to 1.5)
10-14	493/1529 (32)	36/95 (38)	6.8 (4.2 to 9.4)	1.4 (0.6 to 2.2)
15-17	205/1529 (13)	16/95 (17)	7.2 (3.4 to 11.0)	1.5 (0.5 to 2.5)
Child attending				
Day care	335/1537 (22)	19/95 (20)	5.4 (3.3 to 7.4)	1 [Reference]
Primary school	686/1537 (45)	39/95 (41)	5.4 (4.1 to 6.6)	1.0 (0.6 to 1.5)
Secondary school	516/1537 (34)	37/95(39)	6.7 (3.8 to 9.6)	1.3 (0.5 to 2.0)
BMI				
Underweight or normal weight	1208/1520 (79)	80/95 (84)	6.2 (4.9 to 7.5)	1 [Reference]
Overweight or obesity	312/1520 (21)	15/95 (16)	4.6 (2.3 to 6.9)	0.7 (0.4 to 1.1)
Born premature				
No	1447/1520 (95)	87/93 (94)	5.7 (4.5 to 6.9)	1 [Reference]
Yes (<37 wk)	73/1520 (5)	6/93 (6)	7.6 (1.9 to 13.3)	1.3 (0.3 to 2.4)
Chronic medical conditions				
None	1427/1525 (94)	90/95 (95)	5.9 (4.7 to 7.2)	1 [Reference]
Asthma	48/1525 (3)	4/95 (4)	7.4 (0.5 to 14.3)	1.3 (0.1 to 2.4)
Other conditions	50/1525 (3)	1/95 (1)	2.0 (−2.0 to 6.1)	0.3 (−0.4 to 1.0)
Neighborhood				
West Island	487/1537 (32)	17/95 (18)	3.4 (2.4 to 4.3)	1 [Reference]
Mercier-Hochelaga-Maisonneuve	329/1537 (21)	28/95 (29)	7.8 (5.2 to 10.5)	2.3 (1.3 to 3.4)
Montreal North	215/1537 (14)	22/95 (23)	9.3 (6.4 to 12.2)	2.8 (1.6 to 3.9)
Plateau-Mont-Royal	506/1537 (33)	28/95 (29)	5.3 (3.5 to 7.0)	1.6 (0.9 to 2.3)
Parental respondent’s level of education				
<Bachelor’s degree	366/1517 (24)	18/95 (19)	4.7 (2.6 to 6.8)	1 [Reference]
Bachelor’s degree	606/1517 (40)	41/95 (43)	6.3 (4.5 to 8.2)	1.4 (0.8 to 2.0)
≥Master’s degree	545/1517 (36)	36/95 (38)	6.2 (4.2 to 8.2)	1.3 (0.6 to 2.1)
Parental respondent’s race and ethnicity				
Racial or ethnic minority	179/1512 (12)	22/95 (23)	11.0 (6.9 to 15.1)	2.1 (1.1 to 3.1)
White	1333/1512 (88)	73/95 (77)	5.2 (3.9 to 6.4)	1 [Reference]
Adults in household, No.				
1	182/1526 (12)	10/95 (11)	5.2 (1.8 to 8.6)	1 [Reference]
2	1188/1526 (78)	70/95 (74)	5.6 (4.2 to 6.9)	1.1 (0.3 to 1.8)
≥3	156/1526 (10)	15/95 (16)	8.8 (4.7 to 12.9)	1.7 (0.4 to 3.0)
Children in household, No.				
1	326/1526 (21)	24/95 (25)	6.9 (3.7 to 10.1)	1 [Reference]
2	834/1526 (55)	44/95 (46)	5.0 (3.6 to 6.5)	0.7 (0.3 to 1.1)
≥3	366/1526 (24)	27/95 (28)	6.9 (4.3 to 9.4)	1.0 (0.4 to 1.6)
Bedrooms in household, No.				
1-2	314/1526 (21)	18/95 (19)	5.4 (3.2 to 7.7)	1 [Reference]
3-4	1112/1526 (73)	73/95 (77)	6.2 (4.8 to 7.5)	1.1 (0.6 to 1.6)
≥5	100/1526 (7)	4/95 (4)	3.8 (0 to 7.9)	0.7 (−0.1 to 1.5)
Any household member working in				
Other occupations	869/1526 (57)	44/95 (46)	4.8 (3.5 to 6.2)	1 [Reference]
Essential occupation other than health care	132/1526 (9)	17/95 (18)	6.8 (3.8 to 9.8)	1.4 (0.7 to 2.2)
Health care	525/1526 (34)	34/95 (36)	7.9 (5.0 to 10.7)	1.6 (0.8 to 2.4)
Household member has underlying health issues that may put them at higher risk if exposed to COVID-19				
No	1275/1526 (84)	83/95 (87)	6.2 (4.9 to 7.5)	1 [Reference]
Yes	251/1526 (16)	12/95 (13)	4.6 (1.3 to 7.9)	0.7 (0.2 to 1.3)
Child had positive COVID-19 diagnosis via RT-PCR prior to serology testing^a^				
No				
Child was not tested	829/1535 (54)	48/95 (51)	5.5 (4.2 to 6.7)	1 [Reference]
All tests negative	697/1535 (45)	30/95 (32)	4.1 (2.7 to 5.5)	0.8 (0.4 to 1.1)
Yes, at least 1 positive test	9/1535 (1)	17/95 (18)	65.4 (44.9 to 85.9)	11.9 (7.7 to 16.2)
Household member other than child had confirmed COVID-19 infection via RT-PCR				
No	1475/1526 (97)	71/95 (75)	4.6 (3.6 to 5.6)	1 [Reference]
Yes	51/1526 (3)	24/95 (25)	32.0 (19.9 to 44.1)	7.0 (3.9 to 10.0)

Abbreviations: BMI, body mass index; NA, not applicable; RT-PCR, reverse transcriptase–polymerase chain reaction.

^a^ Median time between RT-PCR positive test and serology test was 109 days (IQR, 66-146 days).

Table 2 shows adjusted seroprevalence differences and ratios for type of establishment, household occupations, parental race and ethnicity, and neighborhood. With adjustment for sex, age, and timing of DBS, there were no longer significant differences in seroprevalence rates by neighborhood. The differences in seroprevalence between day care, primary school, and secondary school aged children remained insignificant, with secondary school aged children having the highest seroprevalence at 6.7% (95% CI, 4.6%-8.8%). Having a household member working in health care was consistent with increased seroprevalence (adjusted seroprevalence ratio, 1.5%; 95% CI, 0.8%-2.3%). The children of parents from racial and ethnic minority groups were more likely to be seropositive compared with children of White parents (adjusted prevalence ratio, 1.9%; 95% CI, 1.1%-2.8%).

**Table 2.** Adjusted Seroprevalence Differences and Ratios by Type of Establishment, Parental Education, and Parental Race and Ethnicity, October 2020 to March 2021 in Montreal, Canada

Category	Adjusted seroprevalence (95% CI)	Adjusted seroprevalence	
		Difference (95% CI)	Ratio (95% CI)
Neighborhood^a^			
West Island	4.8 (2.8 to 6.8)	0 [Reference]	1 [Reference]
Mercier-Hochelaga-Maisonneuve	6.2 (3.8 to 8.6)	1.3 (−2.3 to 5.2)	1.3 (0.4 to 2.2)
Montreal North	7.3 (4.5 to 10.1)	2.5 (−1.5 to 6.5)	1.5 (0.5 to 2.5)
Plateau-Mont-Royal	5.4 (3.2 to 7.6)	0.6 (−2.3 to 3.6)	1.1 (0.5 to 1.8)
Child attending^b^			
Day care	5.0 (3.0 to 7.0)	0 [Reference]	1 [Reference]
Primary school	5.6 (4.3 to 6.9)	0.6 (−1.9 to 3.1)	1.1 (0.6 to 1.7)
Secondary school	6.7 (4.6 to 8.8)	1.7 (−1.4 to 4.7)	1.3 (0.6 to 2.1)
Any household member working in^c^			
Other occupations	4.9 (3.5 to 6.4)	0 [Reference]	1 [Reference]
Essential occupation other than health care	6.7 (4.3 to 9.1)	1.7 (−1.3 to 4.8)	1.4 (0.7 to 2.0)
Health care	7.5 (4.8 to 10.4)	2.6 (−0.5 to 5.8)	1.5 (0.8 to 2.3)
Parental respondent’s race and ethnicity^c^			
Racial or ethnic minority group	10.2 (6.5 to 13.9)	4.9 (1.0 to 8.8)	1.9 (1.1 to 2.8)
White	5.3 (4.2 to 6.3)	0 [Reference]	1 [Reference]

^a^ Adjusted for age, sex, and date of dried blood spot.

^b^ Adjusted for sex, neighborhood, and date of dried blood spot.

^c^ Adjusted for age, sex, neighborhood, and date of dried blood spot.

Our study population was more educated, with fewer participants from racial and ethnic minority groups compared with neighborhood characteristics of adults from the 2016 census. When standardizing our seroprevalence estimate of 5.8% (95% CI, 4.6%-7.0%) to the race and ethnicity distribution of the 2016 census, it increased slightly to 6.2% (95% CI, 5.3%-7.1%).

**Figure 2.**
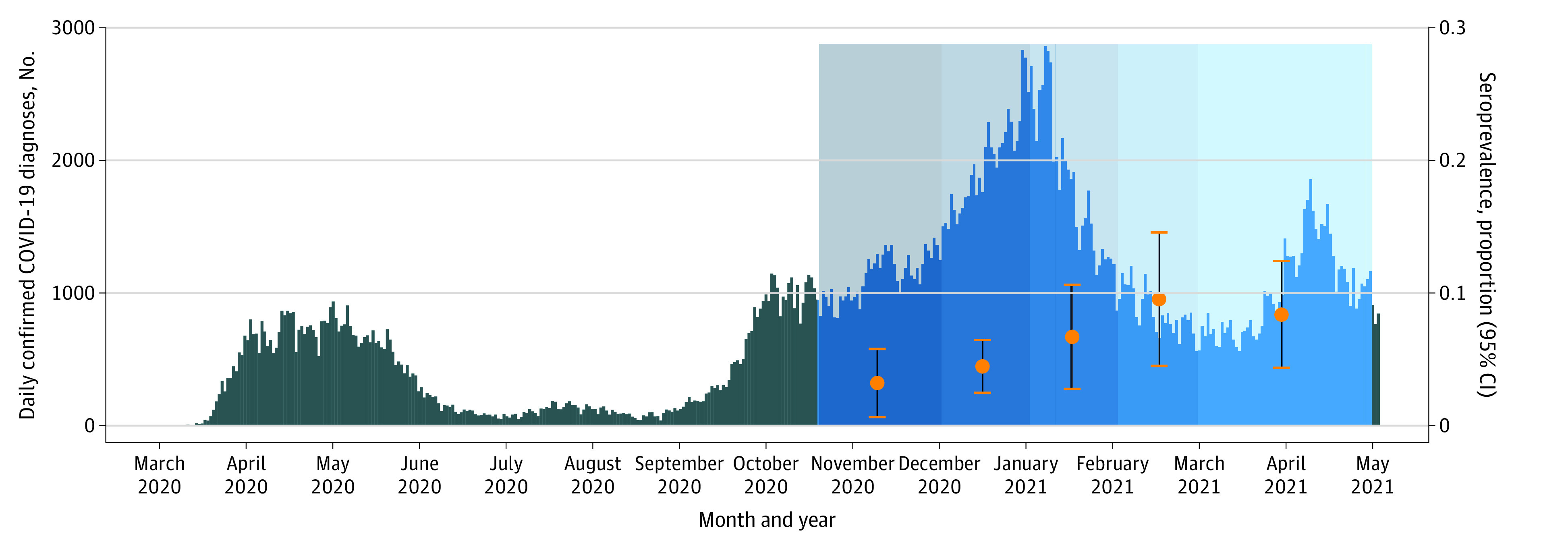
Estimated Seroprevalence for Participants Compared With Overall Daily Confirmed COVID-19 Cases in Quebec The dots and whiskers indicate seroprevalence estimates and 95% CIs for the study population for different months during the first wave of data collection. The shaded area marks the study period, with the gradient indicating different months.

Seroprevalence estimates by month of DBS collection (presented alongside confirmed daily COVID-19 case numbers for the province of Quebec) suggest an increasing trend over time, from 3.2% (95% CI, 0.7%-5.8%) in October to November 2020 to 8.4% (95% CI, 4.4%-12.4%) in March to April 2021 (Figure 2). These estimates are adjusted for age, sex, and neighborhood (to account for differential timing of recruitment across the neighborhoods). Finally, crude seroprevalence rates were calculated for different prevention practices that parents reported for behaviors at the household level. No practices were significantly associated with the serological status of children (eFigure 2 in the Supplement).

## Discussion

The baseline results of our seroprevalence study provide important insight into the proportion of children who have detectable levels of SARS-CoV-2 antibodies from one of Canada’s most affected cities during the first (February to August 2020) and second (September 2020 to March 2021) waves of the COVID-19 pandemic.^6^ A particular strength of our study is the selection of schools and day cares that represent socioeconomically and ethnically diverse populations that will be observed over time.

The increase in seroprevalence over time was consistent with the confirmed case trend in Quebec, with school-aged children younger than 18 years contributing 19% to the total incidence in the second wave, compared with only 6% in the first wave.^20,21^ Our estimated seroprevalence among children was comparable with seroprevalence among Montreal’s adult population for the first wave (3.2% vs 3.1%) but was lower for the second wave (8.4% vs 13.8%).^22^ In other parts of Canada, seroprevalence estimates for children from the first and second waves have been considerably lower, ranging from 0.3% to 1.6%.^23,24^ Internationally, seroprevalence estimates for children and youth from the first and second waves are quite varied, ranging from 0.6% in Germany,^2^ 4.3% in France,^10^ 2.4% to 22.3% in Switzerland,^3,11^ and 2.4% to 11.2% in the UK,^25,26^ although the trend of increasing seroprevalence over time was consistently found in the cohort studies.^11,26,27^

In our study, seropositivity was associated with racial and ethnic minority status, which is consistent with the results of other seroprevalence studies^26,28,29^ and the disproportionate impact COVID-19 has had on racial and ethnic minority communities.^30,31^ We did not find any significant differences between the age groups nor did we find any significant associations with the risk of seropositivity and sociodemographic characteristics, such as sex and parent’s educational level, which is similar to other studies.^2,25,32^ We found that only 18% of our seropositive participants had previously received positive results from RT-PCR testing. This finding further confirms the value of serology as a tool for SARS-CoV-2 surveillance and, although RT-PCR and antigen tests remain essential for acute infection detection, having a more accurate understanding of previous infections allows us to better understand and manage community transmission.^33^

### Limitations

Our study had some important limitations, including the potential for selection bias in our results. The seroprevalence estimates could be an overestimation if the participating children were more likely to have COVID-19 compared with nonparticipating children. For example, households that believed that their child may have been exposed to COVID-19 may have been more motivated to participate. Participation rates varied substantially among the different day care centers and schools (median [IQR], 10% [5%-20%]) and were associated with an index of school-level socioeconomic status (SES). Only 5% of eligible children participated from schools in the lower half of the SES distribution vs 24% of eligible children from schools in the upper half. We were not able to capture the characteristics of nonparticipating children or why parents did not wish to participate, although we received anecdotal reports that the DBS procedure was a barrier to participation and that the letter of invitation sometimes went unnoticed. Reluctance to participate due to the DBS procedure may have lowered participation rates, thus affecting the precision of our estimates; however, we do not expect this introduced bias, as we have no reason to believe it differed according to the participants’ eventual serology result. Given the challenges of conducting this study during the pandemic, we were also not able to select our sample in such a way as to ensure it was representative of our target population.

Comparing our study population with neighborhood characteristics of adults from the 2016 census, our study population was more educated and fewer were from racial and ethnic minority groups across all neighborhoods (eTable 1 and eTable 2 in the Supplement).^16^ However, when we standardized our seroprevalence estimates to the race and ethnicity distribution of the 2016 census, our seroprevalence estimate increased slightly to 6.2% (95% CI, 5.3%-7.1%). We also used research-based ELISA with a 95% sensitivity, which is in line with sensitivity estimates of currently available commercial assays,^34^ but which nonetheless may have led to a small number of false negatives. Furthermore, there were 9 children with positive RT-PCR tests prior to the DBS collection, as reported by their parents, who had a negative serology result. These children were largely asymptomatic compared with children who were seropositive and had positive RT-PCR test results, likely causing a weaker immune response to SARS-CoV-2.^35,36^ Among children who reported at least 1 RT-PCR test prior to providing the DBS sample, the length of time between the most recent RT-PCR test date and DBS date did not significantly differ between seropositive and seronegative children. We may also have not detected children who were previously infected with the virus as antibody levels can wane over time.^37,38^

## Conclusions

Our results provide evidence of the seroprevalence status of Canadian children and highlight the benefits of serological testing in children for SARS-CoV-2. They also support the observation that children experience fewer symptoms from infection with the virus than adults,^4,39^ although they represent a potential risk to their households and communities.^40,41^ Contributing to further evidence of COVID-19 inequities, our results were consistent with higher rates of seropositivity among racial and ethnic minority groups. It will be important to continue monitoring the serological status of children, particularly in the context of VOC and in the absence of high vaccine coverage.

## Notes

Supplement. **eFigure 1. **Prevalence of Symptoms at Time of RT-PCR Test by Serostatus of Participant **eFigure 2. **Household Protective Behaviors by Serostatus of Child **eTable 1. **Comparison of EnCORE Sample and 2016 Census Distribution for Self-reported Race and Ethnicity and Education Level (Adult Population Aged 0-65 Years) and Neighborhood of Residence **eTable 2.** Comparison of EnCORE Adjusted Seroprevalence Estimates Standardized to the Covariate Distributions of 2016 Census Data on Race and Ethnicity and Neighborhood of Residence
