# Supplementary material for: Seroprevalence of SARS-CoV-2 Antibodies Among Children in School and Day Care in Montreal, Canada
Source: JAMA Netw Open. 2021 Nov 23;4(11):e2135975. doi: 10.1001/jamanetworkopen.2021.35975 (PMC8611475; doi:10.1001/jamanetworkopen.2021.35975)
Supplement: Supplement. — eFigure 1. Prevalence of Symptoms at Time of RT-PCR Test by Serostatus of Participant eFigure 2. Household Protective Behaviors by Serostatus of Child eTable 1. Comparison of EnCORE Sample and 2016 Census Distribution for Self-reported Race and Ethnicity and Education Level (Adult Population Aged 0-65 Years) and Neighborhood of Residence eTable 2. Comparison of EnCORE Adjusted Seroprevalence Estimates Standardized to the Covariate Distributions of 2016 Census Data on Race and Ethnicity and Neighborhood of Residence [file jamanetwopen-e2135975-s001.pdf]

Supplemental Online Content

Zinszer K, McKinnon B, Bourque N, et al. Seroprevalence of SARS-CoV-2 antibodies among children in school and day care in Montreal, Canada. *JAMA Netw Open.* 2021;4(11):e2135975. doi:10.1001/jamanetworkopen.2021.35975

eFigure 1. Prevalence of Symptoms at Time of RT-PCR Test by Serostatus of Participant

eFigure 2. Household Protective Behaviors by Serostatus of Child

eTable 1. Comparison of EnCORE Sample and 2016 Census Distribution for Self-reported Race and Ethnicity and Education Level (Adult Population Aged 0-65 Years) and Neighborhood of Residence

eTable 2. Comparison of EnCORE Adjusted Seroprevalence Estimates Standardized to the Covariate Distributions of 2016 Census Data on Race and Ethnicity and Neighborhood of Residence

This supplemental material has been provided by the authors to give readers additional information about their work.

© 2021 Zinszer K et al. *JAMA Network Open.*

eFigure 1. Prevalence of Symptoms at Time of RT-PCR Test by Serostatus of Participant

Grey bars indicate negative RT-PCR tests (n=881) and blue bars indicate positive RT-PCR tests (n=28) reported by participants. The lines indicate 95% CI.

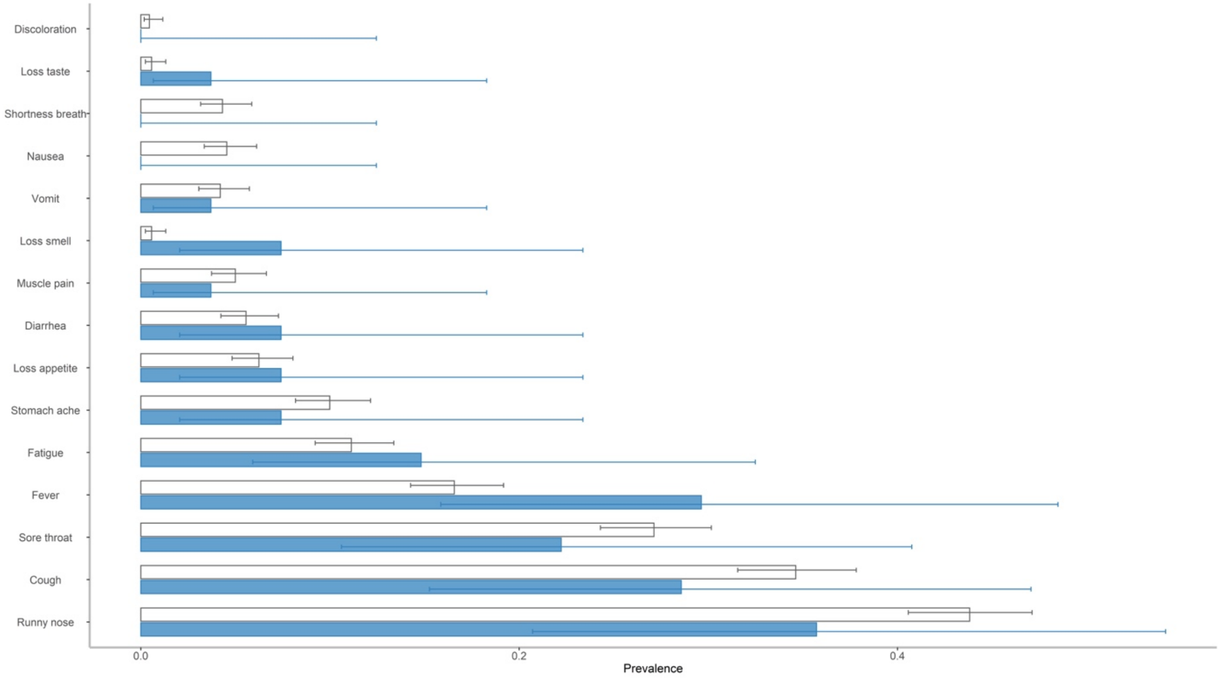

© 2021 Zinszer K et al. *JAMA Network Open.*

eFigure 2. Household Protective Behaviors by Serostatus of Child

Grey bars indicate negative seronegative participants and blue bars indicate seropositive participants. The lines indicate 95% CI.

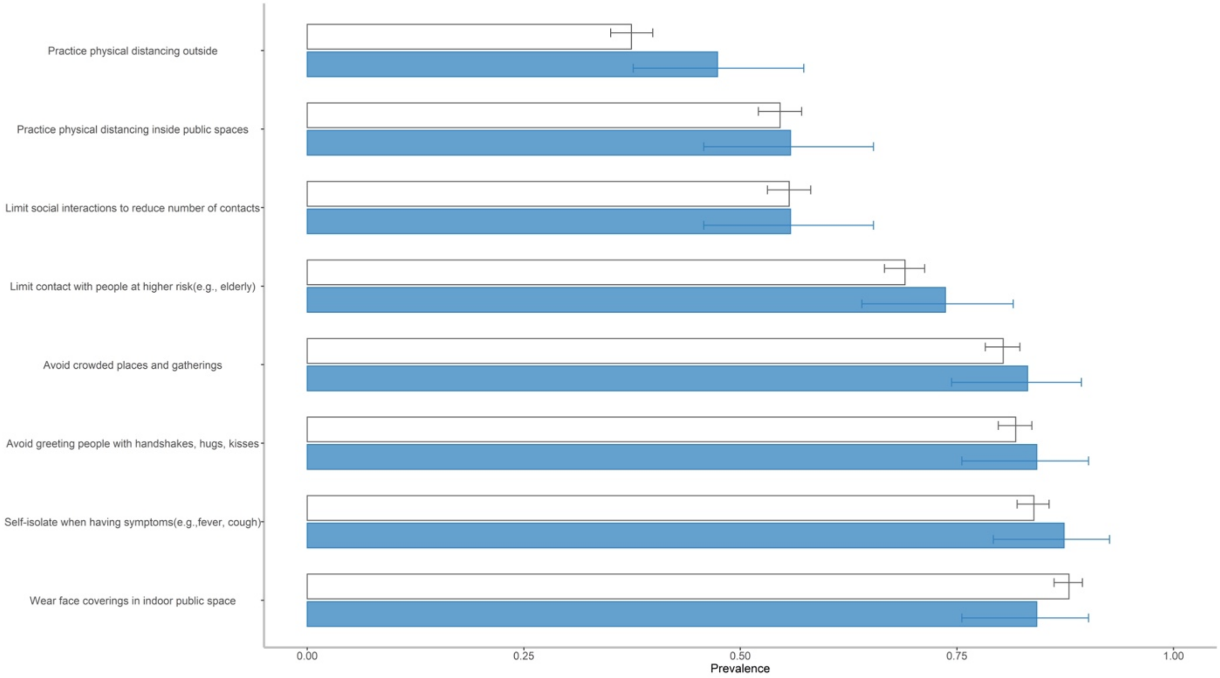

© 2021 Zinszer K et al. *JAMA Network Open.*

eTable 1. Comparison of EnCORE Sample and 2016 Census Distribution for Self-reported Race and Ethnicity and Education Level (Adult Population Aged 0-65 Years) and Neighborhood of Residence

	**EnCORE sample (%)**	**Census distribution for a population-weighted sample for 4 EnCORE neighbourhoods**
Parental respondent's race/ethnicity		
Not a visible minority	87.6	73.2
Visible minority	12.4	26.8
Neighbourhood		
West Island	30.9	31.4
Mercier-Hochelaga-Maisonneuve	21.9	22.0
Montreal North	14.5	17.8
Plateau Mont-Royal	32.7	28.8

© 2021 Zinszer K et al. *JAMA Network Open.*

eTable 2. Comparison of EnCORE Adjusted Seroprevalence Estimates Standardized to the Covariate Distributions of 2016 Census Data on Race and Ethnicity and Neighborhood of Residence

	**Adjusted seroprevalence [a] (95% CI)**
EnCORE population	5.9 (4.9, 6.9)
EnCORE population standardized to the Census visible minority distribution	6.3 (5.2, 7.4)
EnCORE population standardized to the Census visible minority and neighbourhood of residence distributions	6.2 (5.3, 7.1)

[a] Estimates are adjusted for age, sex, neighbourhood, parental race/ethnicity, and date of DBS.

© 2021 Zinszer K et al. *JAMA Network Open.*
